# Effects of diversity in olfactory environment on children’s sense of smell

**DOI:** 10.1038/s41598-018-20236-0

**Published:** 2018-02-13

**Authors:** Lenka Martinec Nováková, Jitka Fialová, Jan Havlíček

**Affiliations:** 1 Department of Anthropology, Faculty of Humanities, Charles University, U Kříže 8, 158 00 Prague 5 – Jinonice, Czech Republic; 2 National Institute of Mental Health, Topolová 748, 250 67 Klecany, Czech Republic; 3 Department of Zoology, Faculty of Science, Charles University, Viničná 7, 128 44 Prague 2, Czech Republic

## Abstract

Diversity in children’s everyday olfactory environment may affect the development of their olfactory abilities and odor awareness. To test this, we collected data on olfactory abilities using the Sniffin’ Sticks and odor awareness with Children’s Olfactory Behaviors in Everyday Life Questionnaire in 153 preschool children and retested them one and a half year later. Parents completed an inventory on children’s exposure to a variety of odors and on their own odor awareness using the Odor Awareness Scale. We controlled for the effects of age and verbal fluency on the children’s performance. We found that the children’s odor identification and discrimination scores differed as a function of parental odor awareness. Although these effects were rather small, they were commensurate in size with those of gender and age. To the best of our knowledge, this study is the first to present evidence that diversity in children’s olfactory environment affects variation in their olfactory abilities and odor awareness. We suggest that future studies consider the long-term impact of perceptual learning out of the laboratory and its consequences for olfactory development.

## Introduction

One of the best-documented phenomena in olfactory research is the great degree of interindividual variability in olfactory performance^1^ and metacognition^2^, also referred to as “odor awareness and reactivity” (hereafter referred to as “odor awareness” for short) or “attitudes towards the sense of smell”. Accounts of how this variability in olfactory perception arises are nonetheless still far from complete. The demographic factor routinely listed in the first place is age^3,4^. However, the passing of time (or age) itself is not what drives developmental changes but can only be employed as a proxy for the actual causal mechanisms. For instance, children’s olfactory abilities mature as they grow up, but this is most likely caused by growing experience with odors and improving linguistic abilities^5,6^, broadening working memory span^7^, improving recognition memory^8,9^, changes in nasal aerodynamics and more effective inhalation of odor stimuli^10^, not age itself. Gender (or sex) is another commonly used proxy for individual differences in olfaction, but simply identifying oneself (or being identified) as male, female, or other can hardly suffice as an explanation. The numerous factors that underlie genotypic and phenotypic sex and gender are involved in a complex interplay, and may or may not converge to allow simplifying classifications^11,12^. Thus, although sex/gender is routinely cited as the second most important demographic factor influencing olfaction, and gender differences in olfaction have been sought by a myriad of researchers^13^, investigations of specific, concrete factors influencing normal olfactory function, some of which may align with the sex/gender classification, seem a more productive approach. These cover, among others, such varied but overlapping and interconnected areas as genetic outfit^14^, nasal anatomy^15^, brain anatomy^16^, respiratory-related physiology^17^, reproductive hormone influences^18^, cognitive functions^19^, crossmodal interactions^20^, and cultural influences^21^.

Curiously enough, one factor that is being rather overlooked when it comes to interindividual differences but is otherwise granted close attention as far as cultural differences are concerned, is diversity in olfactory environments and the formative effect of odor exposure. It is widely acknowledged that differences in olfaction between individuals coming from different cultures stem from long-term, frequent exposure to certain odors within specific contexts^22^. These, in turn, not only come to acquire culturally specific meanings^23^, but may also be perceived as more readily identifiable or categorizable, pleasant, familiar, and more (or less) intense by members of one culture than of another^21,24,25^. Moreover, such variation in experience with odors may result in cross-cultural variation in certain olfactory abilities^26^ and olfactory metacognition^24,27,28^. Cross-cultural differences are one level at which the effects of living in diverse olfactory environments are manifested but they are also sure to give rise to interindividual differences in olfaction *within* a given culture.

The ways in which odor exposure in everyday life shapes an individual’s olfactory abilities and metacognition, i.e. odor awareness, are rather difficult to quantify, hence the scarcity of studies on this topic. The rationale behind this line of inquiry are the effects of perceptual learning, a phenomenon whereby sensory experience brings about changes in brain function and behavior^29–31^. Perceptual learning can occur by means of training, i.e. directed, focused program of instruction, or experience, which consists in unstructured exposure to a wide variety of stimuli^32^. Due to methodological problems with assessment of variation in everyday olfactory environments, most evidence comes from studies with untrained individuals who underwent a specific olfactory training program in the laboratory and from those with experts, mostly perfumers and wine tasters. Altogether these studies show that the abilities of odor identification and discrimination indisputably benefit from olfactory training/exposure in a significant way^33^. Findings on olfactory sensitivity are, however, rather contradictory^34–36^. On the one hand, repeated exposure to a given odor in some untrained individuals may lead to heightened sensitivity to it^37,38^ as well as to alleviation of specific anosmia in the case of the 16-androstenes^39,40^. The evidence on the effect of odor expertise on odor awareness is more limited. If it is to be conceptualized as including olfactory imagery capacity^41,42^, this indeed seems higher in fragrance experts than in non-expert controls^43^. Other than that, there is a study showing a link between self-reported exposure to food odors and flavors in childhood and adulthood on the one hand and odor identification and awareness on the other^44^.

We believe that developmental inquiry would particularly benefit from considering the formative potential of odor exposure. The one thing that should make it far more effective (and hence its consequences even more tangible) than any professional training or exposure within laboratory settings is its long-term and pervasive nature. Despite that, it has actually rarely been considered at all. To date, the only two studies in children to employ some assessment of their everyday olfactory environment reported mixed results. In a sample of Namibian 8–15-year-olds, Saxton *et al*.^28^ did not find a significant link between self-reported olfactory environment (as assessed by questions on the number of siblings, number of animals, and cooking frequency) and odor awareness or olfactory abilities. However, as the authors note, the sample size was quite small (N = 52) and more sensitive measures of individual odor exposure may have been needed. These were used in a recent study by Martinec Nováková and Vojtušová Mrzílková^45^, who showed that the degree of preschool children’s odor exposure in everyday contexts, as assessed by their parents, predicted their odor awareness, but not olfactory abilities. Self- or parental reports of what a child’s everyday olfactory environment smells like is one approach to assess odor exposure. Another, not yet employed in previous studies, is information on those who participate in shaping it, especially the parents. Of course, certain aspects of our olfactory environments and acuity of the sense of smell are non-negotiable and more or less given by the geographic location and place of residence^46,47^ but others can be actively modified through the lifestyles we adopt^48–53^. Behaviors and lifestyles may be transmitted across generations^54–56^ and various aspects of children’s lifestyles are heavily influenced by their parents^57^. Thus, family appears to play the most crucial role not only in familiarization with specific odors, but also in formation of olfaction-related attitudes, practices, and behaviors and so on. Perhaps most notably, this is the case as regards dietary habits^58^ or moralization of certain olfaction-related activities, such as smoking^59^, or odors, such as personal (body) odors^60^. Hence, including information on parental odor awareness may help shed additional light on children’s everyday olfactory environments and how these shape the development of their olfactory abilities and odor awareness.

In the present study, we were interested in how children’s everyday olfactory environment and parental odor awareness affect the development of their olfactory abilities and odor awareness. To achieve this, we collected olfactory data in over 150 preschool children and retested them one and a half year later. In so doing, we controlled for the effects of age and verbal fluency, which were found to modulate children’s olfactory abilities^24,61^ and odor awareness^62^. We hypothesized that children of parents who reported higher self-assessed odor awareness and greater exposure of their offspring to diverse olfactory stimuli would outperform their peers on odor identification and discrimination and also exhibit greater odor awareness, regardless of gender. First, using a categorical principal component analysis, we obtained four components of the children’s odor exposure. Second, due to a fair amount of missing data, imputation of missing values was performed. Finally, using a multivariate repeated measures analysis of variance (MANOVA) model, we tested for the effects of children’s gender (between-subject factor), age at the first session, verbal fluency, four components of odor exposure, and averaged mother’s and father’s odor awareness (covariates) on children’s identification, discrimination, and threshold scores as well as their odor awareness (within-subject variables). We decided to err on the side of caution and performed this analysis on both the imputed dataset and the original one.

## Results

### Effects of diversity of olfactory environment and demographic variables on children’s olfaction – imputed data

A repeated-measures MANOVA showed no significant within-subject differences but a multitude of between-subject ones. As can be seen in detail from Table 1, these were related, as expected, to age, gender, and verbal fluency, but also to the degree of averaged parental odor awareness. Multivariate tests yielded effect sizes of Cohen’s *f* ^2^ ranging between 0.10 and 0.12, except for verbal fluency, for which it was 0.33. According to Cohen^63^ and Murphy *et al*.^64^, the effect sizes of age, gender, and parental odor awareness can be thought of as small to medium, while that of verbal fluency was medium to large. Univariate tests showed that the effects of gender and parental odor awareness pertained to children’s odor identification and discrimination, with Cohen’s *f* ^2^ ranging between 0.04 and 0.09, indicating effects small in size, but still of “practical” significance^65^. Namely, better olfactory identification and discrimination performance was observed in girls and children whose parents reported greater odor awareness. Furthermore, the children’s age at study commencement modestly affected odor identification and threshold, Cohen’s *f* ^2^ = 0.07 and 0.03, respectively, with the older children exhibiting better odor identification and higher sensitivity. Finally, verbal fluency had a medium to large effect on the children’s odor awareness, Cohen’s *f* ^2^ = 0.31. Specifically, the children who produced more words on the verbal fluency test received higher scores in the interview.

**Table 1:** Results of repeated-measures analyses on imputed (N = 153, 76 M) and non-imputed (N = 59, 26 M) data. Shown are multivariate and univariate tests for between-subject effects (multivariate Pillai’s Trace, F, p-value, and Cohen’s *f* ^2^ computed from SPSS-produced partial η^2^). In imputed data, df_1_ and df_2_ for the F-statistic were 4, 141 and 1, 144 for multivariate and univariate tests, respectively, while in non-imputed ones these were 4, 47 and 1, 50, respectively. OE1 = Food diversity and aroma, OE2 = Engagement in cooking and household chores, OE3 = Scent intensity, and OE4 = Edible odors, awareness, and naming. Significant effects (p < 0.05) are highlighted in italics.

	Imputed data								Non-imputed data							
	Gender	Age	Verbal fluency	Parental OA	OE 1	OE 2	OE 3	OE 4	Gender	Age	Verbal fluency	Parental OA	OE 1	OE 2	OE 3	OE 4
**Multivariate tests**																
Pillai’s Trace	0.087	0.102	0.249	0.105	0.043	0.016	0.034	0.016	0.193	0.135	0.299	0.061	0.374	0.105	0.079	0.002
F	3.363	3.990	11.679	4.150	1.584	0.590	1.233	0.579	2.809	1.829	5.007	0.762	7.015	1.378	1.329	0.020
p	*0*.*01*2	*0*.*004*	<*0*.*001*	*0*.*003*	0.182	0.670	0.300	0.678	*0*.*036*	0.139	*0*.*00*2	0.555	<*0*.*001*	0.256	0.415	0.999
Cohen’s *f* ^2^	0.095	0.114	0.332	0.117	0.045	0.016	0.035	0.016	0.239	0.156	0.427	0.065	0.597	0.117	0.086	0.002
**Univariate tests**																
**Identification**																
F	8.013	9.991	0.271	5.648	2.832	0.184	2.489	0.106	6.341	5.734	2.143	0.938	13.913	0.473	0.833	0.001
p	*0*.*005*	*0*.*00*2	0.604	*0*.*019*	0.095	0.669	0.117	0.745	*0*.*015*	*0*.*020*	0.149	0.337	<*0*.*001*	0.495	0.366	0.982
Cohen’s *f* ^*2*^	0.056	0.070	0.002	0.040	0.019	<0.001	0.017	0.001	0.127	0.115	0.043	0.018	0.279	0.009	0.016	<0.001
**Discrimination**																
F	6.345	2.961	2.500	12.932	0.606	1.600	2.612	1.017	5.679	0.002	1.536	2.793	2.014	1.412	3.700	0.021
p	*0*.*013*	0.087	0.116	<*0*.*001*	0.438	0.208	0.108	0.315	*0*.*021*	0.968	0.221	0.101	0.162	0.240	0.060	0.886
Cohen’s *f* ^*2*^	0.044	0.020	0.017	0.089	0.004	0.011	0.018	0.007	0.114	<0.001	0.031	0.056	0.041	0.028	0.074	<0.001
**Threshold**																
F	1.890	4.959	0.027	0.396	0.931	0.090	0.068	1.325	0.099	0.043	0.002	0.165	0.043	<0.001	0.016	0.017
p	0.171	*0*.*028*	0.869	0.530	0.336	0.765	0.794	0.252	0.754	0.837	0.962	0.686	0.837	0.998	0.901	0.897
Cohen’s *f* ^*2*^	0.013	0.034	<0.001	0.003	0.006	<0.001	<0.001	0.009	0.002	<0.001	<0.001	0.003	0.001	<0.001	<0.001	<0.001
**COBEL**																
F	0.329	0.006	43.975	1.767	2.560	0.850	1.293	0.028	3.573	2.158	19.154	0.169	13.305	1.564	0.010	0.051
p	0.567	0.940	<*0*.*001*	0.186	0.112	0.358	0.257	0.868	0.065	0.148	<*0*.*001*	0.683	<*0*.*001*	0.217	0.922	0.822
Cohen’s *f* ^*2*^	0.002	<0.001	0.305	0.012	0.017	0.006	0.009	<0.001	0.072	0.043	0.383	0.003	0.266	0.031	<0.001	0.001

### Effects of diversity of olfactory environment and demographic variables on children’s olfaction – non-imputed data

**Figure 1:**
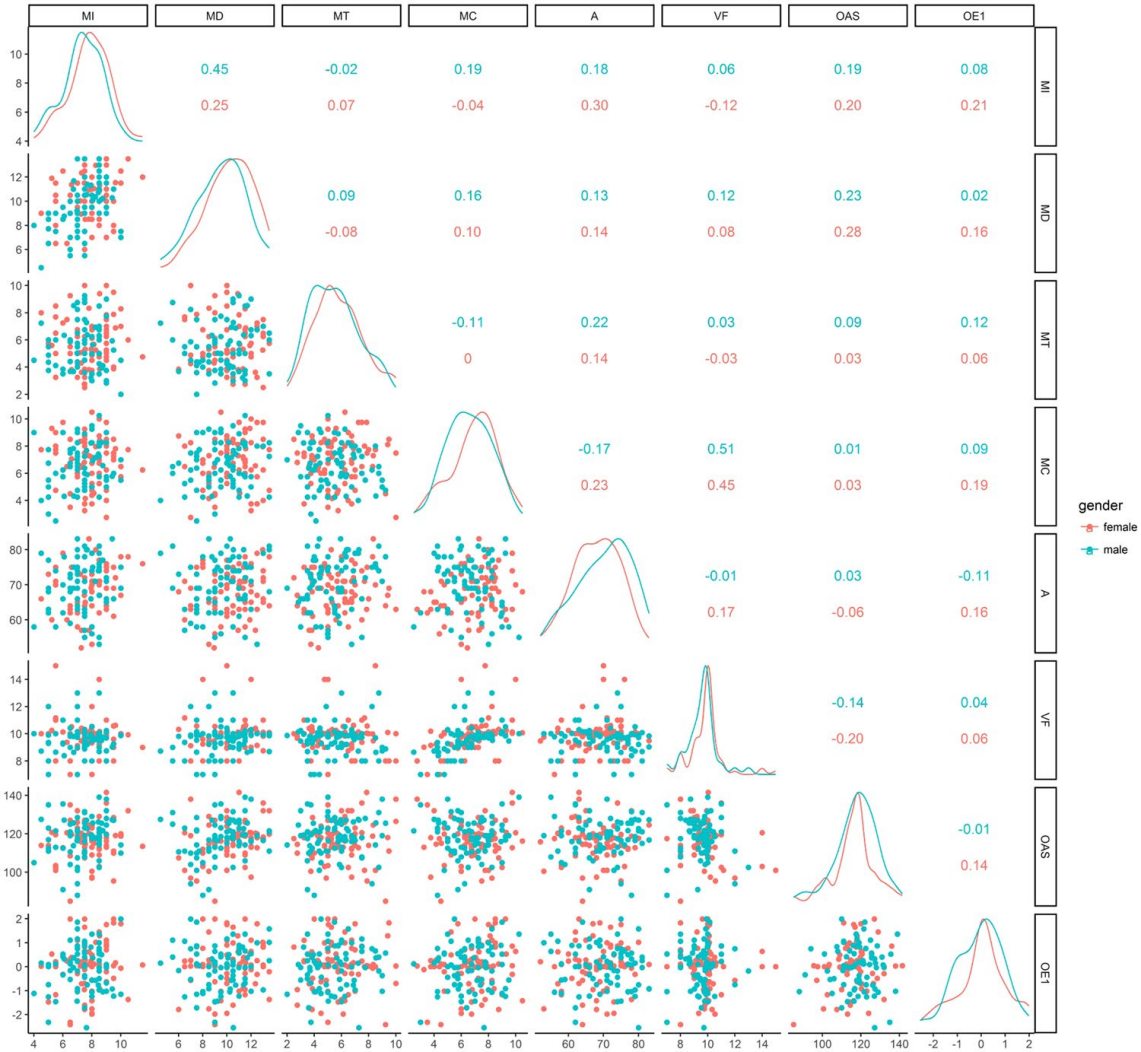
Matrix showing scatter plots of olfactory measures, age, verbal fluency, parental odor awareness, and odor exposure component 1 in the lower diagonal, their distributions on the diagonal and Pearson’s correlations written in the upper diagonal in boys (N = 76) and girls (N = 77) in imputed data. MI = mean identification (mean for the 1^st^ and 2^nd^ testing occasion), MD = mean discrimination, MT = mean threshold, MC = mean total COBEL score, A = age at study commencement, VF = verbal fluency, OAS = parental odor awareness (mean for mothers and fathers), OE1 = first component of odor exposure.

As can also be seen from Table 1, analyses on non-imputed data (N = 59, 26 boys) yielded seemingly slightly different results. Although this analysis did not show any significant effect of repeated measures or interactions involving them, either, it differed from the previous one in that the effect of age and parental odor awareness waned in terms of the formal level of significance, while that of the first component of odor exposure, named “Food diversity and aroma”, appeared. However, as statistical significance largely depends on sample size^66^, which was sub-optimal in the case of non-imputed data, it is advisable to focus on an interpretation of the effect sizes instead. A quick look at Table 1 and comparison of Fig. 1 with Supplementary Figure S1 will reveal that the relationship with parental odor awareness remained the same, while the effects of gender and age increased slightly from small to medium and that of verbal fluency from medium bordering on large to large. The only large effect, larger even than those of the demographic variables, was that of the first component of odor exposure. Namely, children exposed to more diverse food-related odors, as assessed by their parents, scored higher on odor identification and exhibited greater odor awareness. Inspection of the other, non-significant effects will confirm that they were not dramatically different from those in the imputed data, either. Hence, even though the results on the non-imputed data should be regarded with caution because of the small sample size, in fact there was little difference from those obtained on the imputed data in terms of effect sizes.

## Discussion

In the present study, we expected that children’s olfactory abilities, particularly odor identification and discrimination, and odor awareness, would be influenced by the diversity in their olfactory environments. In line with this hypothesis, we found that the children’s olfaction was affected by environmental influences, whether conceptualized in terms of parental odor awareness (identification and discrimination, imputed data, small effects) or parental assessments of the children’s olfactory environments (identification and odor awareness, non-imputed data, medium effects). Besides this, we also replicated some of the standard findings in the literature on the development of olfactory abilities and odor awareness. Namely, odor identification and discrimination scores were on average higher in girls and the former were also higher in older children, as was olfactory sensitivity. These effects were fairly small but still of “practical” significance. Quite large, on the other hand, was the effect of verbal fluency on the children’s odor awareness.

As expected, parental reports of the children’s odor exposure influenced their offspring’s odor awareness, as was the case in the study by Martinec Nováková and Vojtušová Mrzílková^45^ in preschool children and Nováková *et al*.^44^ in young adults. This was despite the fact that different inventories of odor exposure were used, differing slightly in the former and completely in the latter case, in which, moreover, self-reports were provided and a different measure of odor awareness (Odor Awareness Scale)^67^ was used due to a completely different demographic. Thus, there is a mounting converging evidence that diversity in olfactory environment indeed may play a role in how we regard and use our sense of smell, which cannot be simply explained by the fact that all measures (questionnaires, inventories) employed were completed by the same individuals, and hence the so-called extreme response style^68,69^ might have been involved. Even more intriguing is the possibility that this diversity may actually influence some of the olfactory abilities. As mentioned above, if perceptual learning can bring about tangible results after a relatively brief exposure in the laboratory, in real-life settings with its long-term and pervasive effects, the opposite would actually be quite surprising. Also, in the latter case, and especially in children, powerful influences such as social reinforcement are likely to be at work. For instance, “good behaviour” such as washing hands or flushing the toilet is reinforced with verbal praise by parents and teachers^70^. Through signs of disapproval, advertising that heightens insecurities about bodily odors, and other channels that mediate negative societal views of e.g. perspiration, children come to recognize odors that are unacceptable in social interactions and learn to manage and mask them^71^ and even to moralize them^60^. In young children, specific social influences also promote learning about novel odors and flavors, acting to overcome early food neophobia^72^. These and other examples illustrate the crucial role of social environment in the long-term process of learning about odors, which should make such exposure far more effective than any structured training in the laboratory.

This account is broadly in line with evidence of olfactory performance enhancement following olfactory training in the laboratory^38,73–75^ and in professionals (mainly perfumers, wine and beer experts) as compared to naïve individuals^34,35,76^. Importantly, the effects of practice and experience need not be limited to the behavioral level, i.e. performance on various olfactory tasks, but they also appear to be linked to structural and functional variation in certain brain areas in experts and non-experts alike. This idea is supported by positive associations in healthy naïve individuals between performance on various olfactory tasks and the olfactory bulb volume in adults^16,77^ and children and adolescents^78^, gray matter volume in the right orbitofrontal cortex^16^ as well as cortical thickness of the right medial orbitofrontal cortex, right insula or areas around the central sulcus bilaterally^79^. From these associations it follows that if olfactory function is positively influenced by heightened exposure to a variety of olfactory stimuli over the longer term, so should certain brain regions involved in olfactory processing. Direct evidence of changes in brain activity following olfactory training or odor exposure is nevertheless rather limited. In one of the few studies, Li *et al*.^80^ demonstrated that following aversive Pavlovian conditioning, chiral odor pairs initially smelling the same became perceptually distinct, which was accompanied by spatial divergence of odor activity patterns in the piriform cortex. Increased responses in the posterior piriform and medial orbitofrontal cortices along with improved perceptual differentiation for novel odorants related in odor quality or functional group were also observed after an exposure as short as 3.5 min to a target odorant. Importantly, perceptual differentiation performance was predicted by the magnitude of activation of the orbitofrontal cortex^81^. Differences in activation patterns were also observed between individuals with varying levels of olfactory or flavour experience^82–84^. For instance, in a study with professional and trainee perfumers^83^, higher activation in trainees relative to professionals was observed in the right anterior insula during olfactory mental imagery and in the posterior piriform cortex during this task as well as during passive odor perception, suggesting that the two groups used different strategies to process odors. Duration of practice and hence the level of expertise only affected mental imagery capacities, not passive odor perception: the higher the experience level, the weaker the activation of the right posterior piriform cortex, the left orbitofrontal cortex and the left hippocampus. The authors suggested that experts exhibit enhanced perceptual capacity and so require less effort to mentally imagine odors. Structural changes in the brain in healthy individuals remain somewhat less investigated but seem to parallel the functional ones. To be specific, in perfumers, whether trainees or professionals, there was an increase in gray matter volume in the bilateral gyrus rectus/medial orbital gyrus relative to controls. Furthermore, the left-side volume of gray matter in this orbitofrontal area that surrounds the olfactory sulcus as well as in the anterior piriform cortex was positively correlated with the degree of experience of professional perfumers^84^.

Additional evidence of the effects of odor exposure on the brain comes from animal studies. For instance, in the study by Rochefort *et al*.^85^, proliferation among neural progenitors and their survival in the main olfactory bulb was investigated in mice housed in either a standard or an odor-enriched environment for 40 days. They found that while olfactory conditions did not have any influence on the proliferative activity of progenitor cells in the subventricular zone, they did affect the number of newborn neurons in the main olfactory bulb, which, after three weeks, was roughly doubled in the enriched group compared to the standard one. A finding even more important from the perspective of the present study is that the increase of newborn neurons resulting from environmental enrichment had functional consequences on olfactory behavioral performance. To be specific, in mice exposed to the enriched environment, odor memory held circa four times longer than in the controls. In addition, in the former group, recognition of an odor was not affected by an immediate presentation of a second, distractor odor, as evidenced by the fact that odor-enriched mice spent less time investigating the first odor during repeated presentation despite the exposure to the distractor. In contrast, for the controls, the previously encountered odor was as unfamiliar as the second odor. In another study, Mandairon and colleagues^86^ exposed rats to single odors or to pairs of similar or dissimilar odors for one hour twice a day over 20 days. They reported that while the rats did not spontaneously discriminate similar odor pairs prior to the enrichment period, after that, they were able to do so. This improvement was not specific to the odors they had been exposed to. Hence, there is converging evidence from multiple empirical methods suggesting that olfactory enrichment affects brain structure and function and olfactory performance. Future studies should employ the developmental perspective to elucidate the environmental factors that contribute to enhanced olfactory function.

Nevertheless, an alternative account of why the children of parents who reported high odor awareness outperformed others is that parents interested in the study or those who were high-achieving or careful about their image may have encouraged their children, overtly or covertly, to make an effort and perform well in any circumstances, regardless of the task at hand. Psychophysical tests of olfactory abilities require collaboration on the part of the participant and an ability to adhere to oral instructions and very young children tend to lack the motivation to comply with them^87^. As in the case of school performance and academic achievement, children seen as differing in motivation may be exposed to differential parental motivation practices^88^, which, in turn, may perhaps be linked more to parents’ general tendency to outperform, make a good impression, etc. than olfactory interests. Specifically in terms of the interview with children to gauge their odor awareness, some children may tend to exaggerate their responses about unwanted odors and their control. These children may be encouraged to do so, if unknowingly, by parents who care about the impression they and their children give about socially desirable behaviors in general. Even though the parents or other caregivers were not present during the interview, children who (begin to) realize that (de)odorization is widely encouraged within the Western sociocultural context^89^ may have been eager to share this knowledge with the interviewer. Thus, they may not only or necessarily have lived in an environment which was richer in olfactory stimuli, it might be as well that their parents were very careful about their image in this respect and, if unconsciously, passed this message onto their children. Nevertheless, if this account were to be correct, one would expect a strong, significant association between the parental and children’s odor awareness, which was not the case in the present study (see Supplementary Figure S1).

Among the findings routinely reported in the literature on the development of the sense of smell was the effect of gender on odor identification and discrimination^24,62,90–93^. However, a number of studies nevertheless failed to find it^28,45,61,94–98^. Since statistical significance largely depends on sample size^66^, this discrepancy may be caused by differences in sample size, while the effect, in fact, tends to be quite small across studies. However, one should bear in mind that the terms “sex” and “gender” can be understood in a number of ways, e.g. chromosomal, hormonal or endocrine, gonadal, genital, body-type sex, sex of assignment and rearing, brain sex/gender, social and psychological gender^99–101^. What is more, between-gender differences in olfaction may be obscured by gender (non)conformity or sex-atypicality^102^. Therefore, the focus should shift to concrete factors influencing normal olfactory function which tend to align with the sex/gender classification. As noted in the introduction, the majority of developmental studies nevertheless lend little insight into what the actual causes of these gender/sex differences may be. Some indicate that the female verbal superiority is likely to be one factor in children^5,91,103^ and adults^104^ alike. For instance, Monnery-Patris^5^ reported that the gender effect vanished when verbal proficiency (verbal age and olfactory verbal fluency) was controlled for. Another factor might be gender differences in the capacity to comply with verbal instructions and sustain focus on the task at hand during psychophysical olfactory testing. Martinec Nováková and Vojtušová Mrzílková^94^ hypothesised that girls might be better able to handle the testing format. They indeed found higher self-regulatory capacities in girls compared to boys, as assessed by the children’s parents, i.e. superiority in terms of the ability to voluntarily sustain focus on a task, shift attention from one task to another, initiate action, and inhibit it^105^. Yet, girls did not outperform boys on either of the olfactory tests employed. Also, it has been suggested that females since infancy might be exposed to a greater variety of olfactory stimuli in everyday life through certain olfaction-related activities^44,45^. In children, however, parental reports of odor exposure did not differ depending on whether the child was a boy or a girl and girls did not outperform boys on either of the two identification tests or on the discrimination one^45^. More developmental studies are needed to identify other factors which may help explain the gender difference in olfactory abilities.

Further, odor identification and threshold were mildly influenced by the children’s age at the commencement of the study. This is also routinely reported for odor identification^24,61,97^, even though some studies suggest otherwise^28,45^. In general, tasks which depend on lexical, semantic, or symbolic processes exhibit increments in performance as children get older. Again, it is still unclear precisely what factors drive this development. Several mutually non-exclusive factors will likely be involved, such as growing experience with odors and improving linguistic abilities^5,6^, broadening working memory span^7^, improving recognition memory^8,9^, nasal aerodynamics and more effective inhalation of odor stimuli^10^, etc. However, a comprehensive developmental study taking into consideration multiple factors is still missing.

Finally, we also observed a medium to large positive effect of verbal fluency on the odor awareness scores. This is in line with the findings in the original use of the measure^62^, but not with those in two subsequent studies^45,106^. Differences in variability in children’s odor awareness in the present and the two aforementioned studies cannot be invoked to explain this discrepancy, since it was roughly similar. Verbal fluency was more variable in the slightly older children^45,106^ than here, but not significantly so, as the comparison of standard deviations indicated. Different sample sizes cannot account for this discrepancy, either, since the N for which verbal fluency data was available was actually about twice as large in the previous studies, in which nevertheless no link with children’s odor awareness appeared. Besides, the effect size did not even reach the recommended minimum for social science data to be regarded at least as small, but of “practical” significance^65^. There is thus a possibility worth further exploration in future studies that the relationship between children’s odor awareness on the one hand and verbal capacities on the other perhaps only exists at a certain age and vanishes some time after children start school. This is because there may be other moderating or mediating factors at play, affecting both of these variables, such as being more accustomed to interacting and collaborating with adults in authority (e.g. a teacher, researcher), which is crucial for performance both at school and within research settings.

To the best of our knowledge, this study is the first to present evidence that diversity in children’s olfactory environment affects variation in their olfactory abilities and odor awareness. Although these effects were small to medium at best, they were actually commensurate in size with those of demographic variables standardly reported to influence olfaction, i.e. gender and age. We suggest that future studies consider the long-term impact of perceptual learning out of the laboratory and its consequences for olfactory development.

## Materials and Methods

### Participants

The participants were 153 children of Czech origin (76 boys), mean age at study commencement 5.76 ± 0.60 years, range 4.33–6.92 years, mean interval between sessions 18.86 ± 3.49 months. Namely, there were 17 children younger than 5 years of age, 74 aged between 5 and 6 years of age, and 62 over 6 years of age at the first testing occasion. At the start of the study, we collected data in five public mixed-sex kindergartens in Prague and its suburbs. The kindergartens were attended by children from varied social backgrounds. Kindergarten principals were contacted via telephone, e-mail, and in person to inform them about the planned study. Those who had provided permission to perform the study on the kindergarten’s premises were asked to pass the information on to the teachers, who distributed leaflets to the children’s parents. We kept the e-mail addresses and phone numbers the parents had provided to contact them later with an invitation for their children to take part in a second testing. This time it took place either within the schools’ premises or at our department. Girls and boys did not differ in terms of mean age or age distribution at the first testing, t(151) = 1.90, p = 0.06 (boys: 5.67 ± 0.55 years, girls: 5.85 ± 0.63 years), or at the second one, t(151) = 0.573, p = 0.57 (boys: 7.32 ± 0.71 years, girls: 7.26 ± 0.60 years). Nor was there any difference between boys and girls in the interval between the two sessions, t(151) = 1.50, p = 0.14. Data collection took place in the late spring and early autumn, primarily so as to minimize missing data due to dropout during the influenza season. Therefore, possible seasonal effects on olfactory performance were not taken into account. As can be seen from Table 2, some of the measures were nevertheless missing in some children, which is why N is given for each variable and analysis.

**Table 2:** Mean ± SD, range, and valid N for the repeated measures of age in months, age difference between 1^st^ and 2^nd^ testing, Sniffin’ Sticks TDI (threshold, discrimination, identification), and COBEL (total and its food, social, and environmental component). Further, verbal fluency (2^nd^ testing only), olfaction-related activities (CATPCA object scores for the four factors), and mother’s and father’s odor awareness scores are shown in boys, girls, and the total sample. Shown are also t statistics and degrees of freedom for differences between boys and girls, and their effect sizes expressed as Cohen’s d. Significant findings are highlighted in italics. No corrections have been made for running multiple tests. Please note that the identification test was only comprised of 12 instead of the original 16 items.

		**Boys**				**Girls**				**Total**				**Gender difference**		
	Theoretical range	Mean	SD	Range	N	Mean	SD	Range	N	Mean	SD	Range	N	t(df)	p	d
**First testing**																
Age (months)		70.22	7.57	53–83	76	68.04	6.65	52–83	77	69.12	7.18	52–83	153	−1.90 (151)	0.06	0.31
Threshold	1–16	5.12	2.81	0.75–11.50	67	5.78	3.15	0.75–14	73	5.46	3.00	0.75–14	140	1.32 (138)	0.19	0.22
Discrimination	0–16	8.95	2.46	2–14	76	9.34	2.18	4–14	76	9.14	2.32	2–14	152	1.05 (150)	0.30	0.17
Identification	0–12	7.32	1.82	2–11	76	7.56	1.88	3–11	75	7.44	1.85	2–11	151	0.81 (149)	0.42	0.13
COBEL total	0–15	6.56	2.16	1.50–11	75	6.95	2.06	3–11	74	6.75	2.11	1.50–11	149	1.12 (147)	0.27	0.18
COBEL food	0–3	1.46	0.70	0–2.50	75	1.50	0.73	0–2.50	74	1.48	0.71	0–2.50	149	0.34 (147)	0.73	0.06
COBEL social	0–4	1.53	1.08	0–3.50	75	1.93	1.06	0–4	74	1.73	1.09	0–4	149	*2.24 (147)*	*0.03*	*0.37*
COBEL environmental	0–8	3.57	1.09	1–5.50	75	3.52	1.13	1–6	74	3.54	1.11	1–6	149	−0.26 (147)	0.80	0.05
**Second testing**																
Age (months)		87.85	8.53	69–104	76	87.12	7.23	69–103	77	87.48	7.89	69–104	153	−0.57 (151)	0.57	0.09
Age difference (months)		18.44	3.52	12–26	76	19.28	3.44	12–25	77	18.86	3.49	12–26	153	1.50 (151)	0.14	0.24
Threshold		5.98	1.85	2–10	51	5.82	1.99	2–9.50	57	5.89	1.92	2–10	108	−0.44 (106)	0.66	0.08
Discrimination		10.16	2.54	5–16	62	11.13	2.47	5–15	63	10.65	2.54	5–16	125	*2.16 (123)*	*0.03*	*0.39*
Identification		7.40	1.75	2–11	63	8.18	1.60	5–12	62	7.78	1.72	2–12	125	*2.60 (123)*	*0.01*	*0.47*
COBEL total		6.53	2.03	2.50–11	61	7.02	2.34	2.50–13	62	6.78	2.20	2.50–13	123	1.24 (121)	0.22	0.22
COBEL food		1.59	0.69	0–2.50	61	1.55	0.78	0–2.50	62	1.57	0.74	0–2.50	123	−0.31 (121)	0.76	0.05
COBEL social		1.28	1.04	0–3.50	61	1.57	1.06	0–4	62	1.43	1.06	0–4	123	1.55 (121)	0.12	0.28
COBEL environmental		3.66	1.07	1.50–5.50	61	3.90	1.30	2–7	62	3.78	1.19	1,50–7	123	1.12 (121)	0.27	0.20
Verbal fluency		9.58	1.77	7–13	26	9.85	1.84	7–15	34	9.73	1.80	7–15	60	0.58 (58)	0.56	0.15
1 Food diversity and aroma		−0.04	0.93	−2.55–1.99	76	0.04	1.03	−2.42–1.99	77	0	0.98	−2.55–1.99	153	0.49 (151)	0.63	0.08
2 Engagement in cooking and household chores		−0.21	0.97	−2.19–2.32	76	0.21	0.94	−1.66–2.67	77	0	0.98	−0.2.19–2.67	153	*2.71 (151)*	<*0.01*	*0.44*
3 Scent intensity		0.01	0.97	−2.18–1.92	76	−0.01	0.99	−2.85–2.51	77	0	0.98	−0.2.85–2.51	153	−0.17 (151)	0.87	0.02
4 Edible odors, awareness, and naming		−0.07	1.06	−2.58–2.30	76	0.07	0.88	−2.37–2.23	77	0	0.98	−0.2.58–2.30	153	0.85 (151)	0.40	0.14
Mother’s OAS	32–158	119.35	12.42	91–139	49	120.67	11.49	85–147	54	120.04	11.90	85–147	103	0.56 (101)	0.58	0.11
Father’s OAS	32–158	113.44	12.95	79–137	43	109.77	16.52	77–146	39	111.70	14.78	77–146	82	−1.13 (80)	0.26	0.25

### Ethics Statement

All procedures followed were in accordance with the ethical standards of the responsible committee on human experimentation (institutional and national) and with the Helsinki Declaration of 1975, as revised in 2008 (5). The study has been approved by the IRB of the Faculty of Science, Charles University (Approval No. 2008/4). Written informed consent was obtained from the children’s parents and oral informed consent was provided by the children in the presence of a teacher employed by the school. The children-parents pairs each received 300 CZK (approx. 11 EUR) in compensation.

### Olfactory Measures

#### General Considerations

The Sniffin’ Sticks test^107^, manufactured by Burghart Messtechnik GmbH, was used to assess odor identification, discrimination, and threshold. This is one of the most widely used tests of olfactory performance, based on pen-like odor dispensing devices. The Sniffin’ Sticks test has been widely used by clinicians and researchers across Europe to test olfactory abilities in adults^1^ and children^24,90,95^, including Czech ones^28,45,94,108^.

#### Odor Identification

The 16-item identification test (“blue”, as it is referred to by the manufacturer) consists of odors familiar to the general European population, namely orange, leather, cinnamon, mint, banana, lemon, licorice, turpentine, garlic, coffee, apple, clove, pineapple, rose, anise, and fish (exact chemicals are not specified by the manufacturer). In the original version of the test cued identification is employed, in which participants select the verbal label of the target odor from a candidate list of four alternatives. The resulting score is the sum of correct answers (maximum of 16). In the present study, the test was adapted to children who could not read yet or were only beginning to learn reading. This was done by presenting both the targets and distractors in the form of color pictures instead of verbal labels. The process of adaptation of the tests has been described in full detail in Martinec Nováková and Vojtušová Mrzílková^45^. In short, children had been interviewed about their understanding of both the target and distractor odor sources, upon which images depicting items most frequently associated with the given verbal label were selected. These interviews had also revealed that most children were rather unfamiliar with most of the spices (anise, cinnamon, clove, vanilla), menthol, and turpentine. Thus, items involving these odor sources either as a target or distractor (items 3, 8, 12, and 15) were excluded, resulting in the maximum score of 12 for the identification test, with higher scores indicating better identification performance. Prior to the identification task, the researchers always made sure the children understood what the given picture depicted. Odor presentation (sequence of odors presented, distance from the nostrils, exposure duration, etc.) was carried out in a manner recommended by Hummel *et al*.^107^. The interval between odor presentations was circa 20 seconds.

#### Odor Discrimination

No alterations had to be made to the discrimination test. The test of odor discrimination assesses the degree to which an individual can differentiate between odors in suprathreshold concentrations. The set comprises 16 triplets of odorized pens, of which two are identical, and the participant is asked to indicate the odd one. The odorants used in the test and the order of presentation, which was followed, are given in Hummel *et al*.^107^. Presentations of triplets were separated by circa 20 seconds. The score is the total of correct trials (0–16), with higher scores indicating a better ability of odor discrimination.

#### Odor threshold

No alterations were made to the threshold test, either. The olfactory threshold refers to the minimum concentration of a tested odorant that an individual is able to reliably differentiate from a blank sample. The set employed in the present study consisted of 16 dilution steps of n-butanol (targets), each of which formed a triplet with two blanks. As recommended by Hummel *et al*.^107^, a single-staircase, three-alternative forced-choice (3-AFC) method was used, in which, starting with the lowest concentration (dilution number 16), an ascending (low to high concentration) series of even-numbered triplets was presented, with successful trials prompting another presentation of the same triplet in a random order. Two successful trials in a row marked a turning point; starting with the nearest lower concentration, a descending series of triplets was presented until the child failed to detect the target. This marked a reversal towards the higher concentrations and, starting with the next higher concentration, an ascending series of triplets was presented until two correct trials occurred, marking another reversal. The testing was finished after a total of 7 reversals was reached. The threshold score was computed as the arithmetic mean of the dilution number at the last four reversals. Ranging from 1 to 16, higher scores indicate greater olfactory sensitivity (i.e. lower threshold).

### Verbal fluency

Verbal fluency is known to modulate mainly odour identification and children’s reports by which their odor awareness is gauged^24,28,62^. It was tested using a Czech version of the category verbal fluency test adapted to children^109^. First, a training trial was conducted using the transport category, on which a child was asked to name as many means of transport as possible. Understanding was assured by asking the child what transport meant and by giving an example (e.g. a car). Next, the child was encouraged to name as many animals as possible in 60 seconds, while the answers were being immediately written down by the researcher. Verbal fluency was only conducted at the second testing. The verbal fluency score is the count of the items correctly named (theoretical minimum of 0).

### Questionnaires

#### Children’s Olfactory Behaviors in Everyday Life Questionnaire (COBEL)

The children's odor awareness was assessed by means of an interview based on the Children’s Olfactory Behaviors in Everyday Life (COBEL) questionnaire^62^. Having been originally developed with 6- to 10-year-olds, it consists of 16 questions designed to evaluate self-reported awareness of odors in significant everyday contexts, i.e. food, social, and environmental. Each item was coded on a 3-point scale, rating the child as poorly (0), moderately (0.5), or highly (1) olfaction-oriented in the given situation. Although it was used in a previous study with slightly older Czech children^28^, in this age group it transpired that children did not fully understand the rating format of Item 3 (Senses in nature: “When you walk in nature, what do you prefer?”), which involved ranking the following activities in order of preference: touching, smelling, watching, listening^45,106^. Specifically, most children tended to disregard the items to be ranked and offered their own response, which mostly involved “playing” or “running around”. Therefore, the item was excluded from the interview. Thus, the total COBEL score, computed as a sum of the 15 items, ranged from 0 to 15. In addition to the total score, component scores for food (items 1, 2, and 16), social (items 11, 12, 13, and 14), and environmental odors (items 4, 5, 6, 7, 8, 9, 10, and 15) were computed following previous usage of COBEL^24,28,62^. The theoretical range for food, social, and environmental odors is thus 0 to 3, 0 to 4, and 0 to 8, respectively. The actual ranges are given in Table 2. The amended version of the questionnaire used in this study is enclosed in Supplementary Table S2.

#### Odor Awareness Scale (OAS)

To assess individual differences in odor awareness in parents (both mother and father), the Czech version of the 32-item Odor Awareness Scale^67^ was administered. The teachers were asked to distribute the Odor Awareness Scale only to parents who shared the household with each other and their child. The scale was translated by LMN and back-translation was produced by Jaroslava Varella Valentova. For previous usage in Czech participants see Nováková *et al*.^44^. This is a metacognitive measure to learn about people’s self-assessments of their tendency to notice, pay attention, or attach importance to odors in certain everyday situations, and their knowledge of how olfactory experiences shape their everyday behaviors. Items relate, for instance, to the effect of odors on mood (“When a room has an unpleasant smell, does it influence your mood?”), evocation of memories by odors (“Do odors revive strong or vivid memories in you?”), olfactory distraction (“When you are studying, or concentrated in general, do you get distracted by odors in the environment?”), effect on product purchase (“Suppose you are at a supermarket where it smells bad. Is this a reason for you not to return there?”), approach (“When someone has a pleasant body odor, do you find him or her attractive?”), and avoidance (“You are in a public space sitting close to someone who has an unpleasant smell. Do you look for another seat if possible?”). Thirty of the 32 items are a five-category response format (e.g. “always,” “often,” “sometimes,” “seldom,” and “never”), with greater frequency, degree, or probability scoring more points. The total score is obtained by adding up the scores of the individual items, and can range between 32 and 158, with higher scores indicating greater odor awareness. For subsequent analyses, the mother’s and father’s scores were averaged for each child.

#### Children’s Odor Exposure Inventory

Mothers were also asked to complete an inventory regarding their children’s exposure to odors in various everyday contexts. It was based on the Olfactory Diversity Questionnaire (ODQ)^110^, which provides parental reports of children’s odor exposure and lists items involving activities potentially rich in olfactory stimulation. It has been reported to exhibit a moderate to strong association with children’s both free and cued Sniffin’ Sticks odor identification scores^110^. Items include, for instance, raising the child’s awareness of the surrounding odors, child’s exposure to exotic foods and a variety of herbs and spices, use of various scented products, presence of natural sources of odor (e.g. animals) in the household, smoking, or air pollution in the neighborhood. The complete list of the items is given in Supplementary Table S3. Several items were not included in the analysis on the following grounds. Firstly, as regards the item Childcare (1), only 32% of mothers (N = 47) indicated that their child had ever taken part in such an activity. Out of those, 57% (N = 27) responded that the child only participated in childcare with a very low frequency of several times a month to less than once a month (median = 1). Further, exploratory correlations preceding a categorical PCA (CATPCA) showed that items Frequency of use of cosmetic products (2), those listing products/odor sources/hobbies/pets (items 14 through 17), and Breastfeeding (24) did not correlate with any other item. Since recoding to a yes/no response format or rescaling had no effect, and inclusion of these items in the CATPCA invariably led to a marked drop in the percentage of variance accounted for and a structure of loadings difficult to interpret, these items were omitted from the analysis. Finally, strength of environmental odor (19) and of odor from smoking at home (22, 23), respectively, were excluded from the analysis. In terms of environmental odor, only 31% of mothers indicated that there were objects in the vicinity of their home which were a source of strong odor. Of these, over one fourth (29%) nevertheless thought such an object emanated little to no odor. In most households (83%), the members of the family did not smoke at home. Of those who did smoke at home or had neighbors whose smoking odors penetrated to their household, 96% still perceived little to no odor.

To identify the components underlying the reports of odor exposure and to obtain component scores to be used in the subsequent analyses, we performed a categorical principal component analysis (PCA) using the IBM SPSS categorical PCA (CATPCA) Optimal Scaling option. Of the total of 153 cases, 8 cases, in which the survey had not been completed, were excluded from the analysis. The assumptions of the analysis were met, since an exploration of correlations between the variables entered in the analyses showed that extreme multicollinearity (>0.9) or singularity (=0.0) were not a problem, and all the data were positive integer. Ordinal and nominal variables were scaled as such and the former were discretized by ranking. A variable principal normalization method was selected. Dimensions in solution were determined upon multiple trials to obtain the most interpretable structure of loadings. Specifically, eigenvalues were used as an indication of how many dimensions were needed, following the general rule which states that the eigenvalue for a dimension should be larger than 1 when all variables are either single nominal, ordinal, or numerical^111,112^. Joint plots of the category points showed that the categories of variables were separated by the categorical principal components analysis clearly enough as could be expected when the level was truly ordinal. The plot of the object scores revealed no outliers. A rule of thumb was followed that only loadings sharing at least 15% of their variance with the components (i.e. loadings of about 0.40 or greater) should be considered practically significant and useful for interpretation purposes^111^. This is roughly in accord with another recommendation of Stevens^111^ on component loadings with respect to sample size. Specifically, for N > 140 at α = 0.01, component loadings of about 0.434 or slightly less should be considered statistically significant. The CATPCA yielded four components: (i) Food diversity and aroma, (ii) Engagement in cooking and household chores, (iii) Scent intensity, and (iv) Edible odors, awareness, and naming. The resulting components and loadings are given in Table 3.

**Table 3:** An overview of the components (percentage of total variance), items, and loadings. Items with weak loadings (<0.40) are given in italics at the component on which their loadings have been the highest.

**Component**	**Item**	**Loading**
Food diversity and aroma (16.40%)	7 Strength of aroma	0.681
	6 Seasoning	0.628
	5 Foreign cuisines, exotic or unusual foods	0.549
Engagement in cooking and household chores (15.02%)	12 Household chores	0.695
	4 Participation in meal preparation and cooking	0.643
Scent intensity (11.81%)	3 Strength of scent of cosmetic products	0.632
	13 Strength of scent of household products	0.402
	*11 Feasts*	*0*.*373*
Edible odors, awareness, and naming (9.88%)	10 Home baking	−0.534
	21 Attention to odors	0.493
	8 Herbalism	−0.488
	20 Odor naming	0.483
	*9 Home processing of seasonal produce*	*−0*.*356*

### Procedure

Parents and teachers were instructed to only encourage their children to attend the testing sessions, scheduled between 9 a.m. and 3 p.m., when in good respiratory health. Testing took place in a secluded, well-ventilated room without strong ambient odors. First, children were briefly familiarized with the tasks, which were presented as a game, and ensured that they could stop or quit at any time. The order of the olfactory tests, interview based on COBEL, and verbal fluency test was randomized across children. However, within the olfactory tests, the stimuli were presented in the order recommended by Hummel *et al*.^107^. The sheer number of the various olfactory tests and the interview presented a cognitive load that could only be alleviated by splitting them over two sessions. Therefore, each child was tested on two consecutive days or within a week at the very latest. Each session took circa 30 minutes. The parents were sent the Odor Exposure Inventory and the Odor Awareness Scale to complete them at home, which they returned to the teacher. Parents or teachers were never present in the room during the testing session.

### Statistical Analysis

Analyses were carried out with SPSS 24.0^113^ and R^114^. Normality of the raw data was checked, firstly, by visually examining the individual histograms of all relevant variables, secondly, by producing skewness and kurtosis values and their respective standard errors, from which z-scores were computed and compared to the value of 1.96, as suggested by Field^115^, and, thirdly, with multiple Shapiro-Wilk’s W tests. Except the two threshold measures, the assumption of normality was met. Hence, parametric tests were used. Bivariate exploratory correlations involving interval variables or an interval and a dichotomous one were computed using Pearson product-moment correlations. Those involving two ordinal, dichotomous, or an ordinal and a dichotomous variable were calculated with Spearman’s rho, Phi, and Lambda, respectively. Gender differences were tested with t-tests. The r and t statistics were converted to Cohen’s *d* after Cumming^116^. There was a fair amount of missing data, which would render analysis across repeated measures only possible in 59 children (26 boys). Therefore, an imputation of missing values was performed first. To achieve this, the missForest package^117^ was used, available from the Comprehensive R Archive Network (CRAN) and run in the R^114^. Recommended particularly for conducting multiple imputation of mixed data (numeric and factor variables in one data frame)^118^, it has been compared to other imputation methods and found to have the least imputation error for both continuous and categorical variables and the smallest prediction difference (error)^119^. Default settings were used^117,120^. Subsequent multiple t-tests and Pearson’s product-moment correlations did not show any difference between the original and imputed data in any of the variables or any change in relationships between variables within the imputed data compared to the original data, respectively.

Then, the relationship between children’s olfactory abilities and odor awareness, gender, age at study commencement, components of odor exposure, verbal fluency, and parental odor awareness was analysed using a repeated-measures MANOVA, disregarding the interindividual differences in the interval between the two measurement occasions. This was done because correlations between the repeated measures did not markedly change or turn non-significant after controlling for the difference between the ages at study commencement and completion. This was true for both the imputed and non-imputed data. The COBEL, identification, discrimination, and threshold scores were entered as within-subject variables, gender was treated as a between-subject factor, and the rest (age at the first session, verbal fluency, the four components of odor exposure, and mean parental odor awareness) as covariates. Cohen’s *d* for differences between means was computed after Rosnow and Rosenthal^121^. Partial eta squared produced by SPSS was converted to Cohen’s *f* ^2^ after Cohen^63^; see also IBM Support^122^.

### Data Availability

The dataset generated and analysed during the present study is available in the Open Science Framework: osf.io/qhsw7.

## Electronic supplementary material

Supplementary Information
