# Supplementary material for: Effects of diversity in olfactory environment on children’s sense of smell
Source: Sci Rep. 2018 Feb 13;8:2937. doi: 10.1038/s41598-018-20236-0 (PMC5811485; doi:10.1038/s41598-018-20236-0)
Supplement: Supplementary file 1 — Supplementary Information [file 41598_2018_20236_MOESM1_ESM.pdf]

Effects of diversity in olfactory environment on children's sense of smell

Lenka Martinec Nováková[1,2*], Jitka Fialová[2,3], Jan Havlíček[2,3]

[1] *Department of Anthropology, Faculty of Humanities, Charles University, U Kříže 8, 158 00 Prague 5 – Jinonice, Czech Republic*

[2] *National Institute of Mental Health, Topolová 748, 250 67 Klecany, Czech Republic*

[3] *Department of Zoology, Faculty of Science, Charles University, Viničná 7, 128 44 Prague 2, Czech Republic*

[*] Corresponding Author

Correspondence to be sent to: Lenka Martinec Nováková, Topolová 748, 250 67 Klecany, Czech Republic; lenka.novakova@nudz.cz or lenka.novakova@fhs.cuni.cz

Supplementary Figure S1. Matrix showing scatter plots of olfactory measures, age, verbal fluency, parental odor awareness, and odor exposure component 1 in the lower diagonal, their densities on the diagonal and Pearson's correlations written in the upper diagonal in boys (N = 76) and girls (N = 77) in non-imputed data. MI = mean identification (mean for the 1st and 2nd testing occasion), MD = mean discrimination, MT = mean threshold, MC = mean total COBEL score, A = age at study commencement, VF = verbal fluency, OAS = parental odor awareness (mean for mothers and fathers), OE1 = first component of odor exposure.

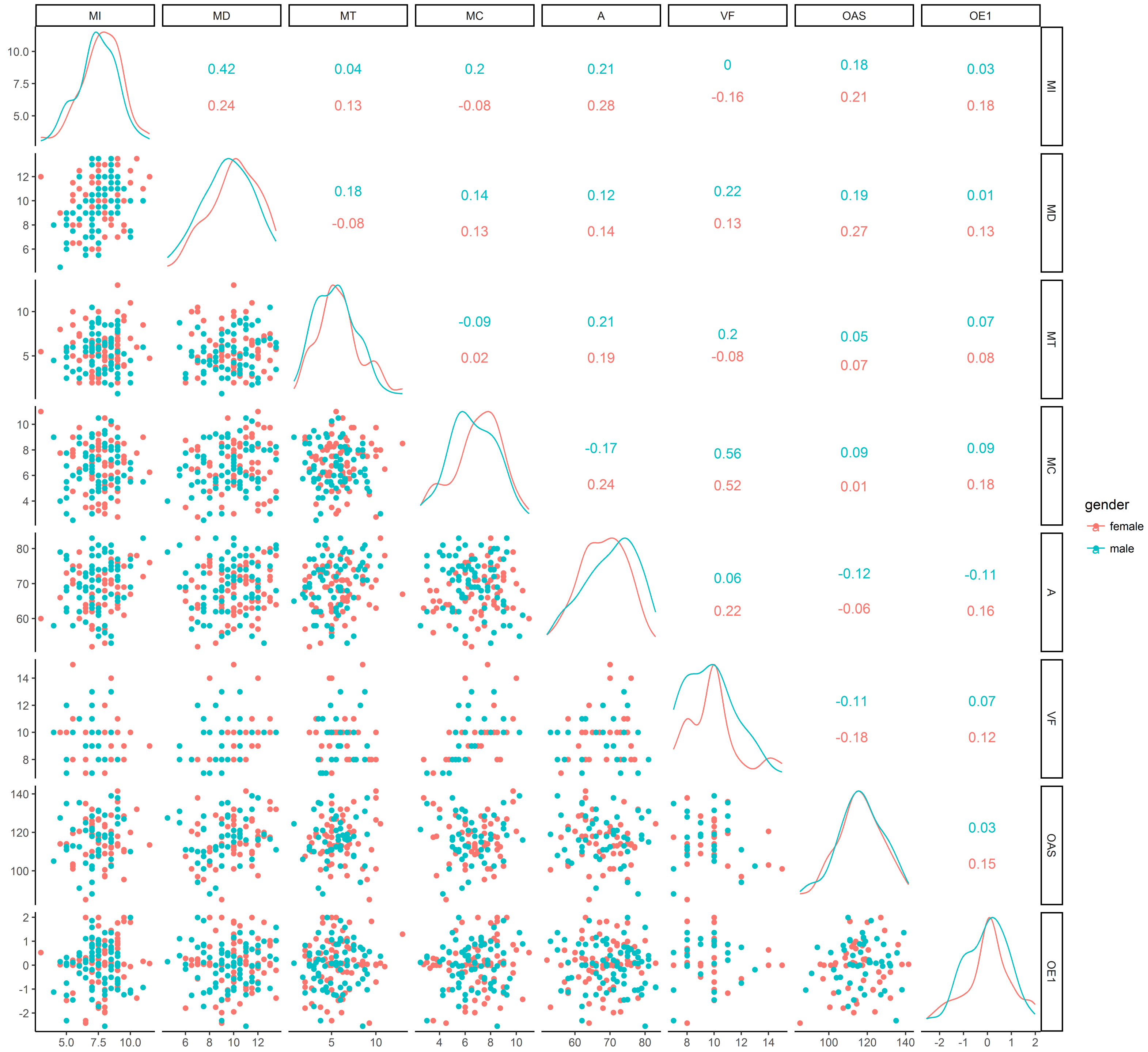

MI
MD
MT
MC
A
VF
OAS
OE1
0.42
0.24
0.04
0.13
0.2
-0.08
0.21
0.28
0
-0.16
0.18
0.21
0.03
0.18
0.18
-0.08
0.14
0.13
0.12
0.14
0.22
0.13
0.19
0.27
0.01
0.13
-0.09
0.02
0.21
0.19
0.2
-0.08
0.05
0.07
0.07
0.08
-0.17
0.24
0.56
0.52
0.09
0.01
0.09
0.18
0.06
0.22
-0.12
-0.06
-0.11
0.16
-0.11
-0.18
0.07
0.12
0.03
0.15
gender
female
male

Supplementary Table S2. Content and scoring method of the 15 items of the COBEL questionnaire [1] used in the present study (Czech version available upon request). The excluded item (Item 3 – Senses in nature) is given in italics.

Item	Wording	Scoring
Food-related olfactory contexts		
Item 1 – Odor in food dislikes	Are there some foods/drinks that you hate (yes/no)? Which ones (up to 6)? For which reasons (for each cited food)?	>2/3 of the reported food items are disliked because of the flavor/odor (1), between 1/3 and 2/3 (0.5), <1/3 (0)
Item 2 – Response to unknown food	Imagine your parents present you a dish you do not know: will you do something before putting it in your mouth (yes/no)? What do you do? Will you smell it (yes/no)?	The child reports spontaneously smelling the unknown food (1), not spontaneously (0.5), reports not smelling the food (0)
Item 16 – Guessing food odor	When you smell a food odor, do you try to guess for fun what it is (never/sometimes/often)?	Often (1), sometimes (0.5), never (0)
Social-related olfactory contexts		
Item 11 – Family odors	Do you find that your parents/siblings smell of something? Imagine they would not smell of anything anymore: would you not care/would it bother you/would it suit you – a little/a lot? Why?	Bothers or suits the child a lot (1), a little (0.5), the child does not care about it or finds that parents smell nothing (0)
Item 12 – People's natural odor	Do you find that people smell of something, even without perfume or deodorant (no/yes some people/yes everyone)?	Everyone (1), some people (0.5), no (0)
Item 13 – Smelling clothes	Do you happen to smell your clothes (never/sometimes/often)? Why?	Often (1), sometimes (0.5), never (0)
Item 14 – Smelling self-odor	Do you happen to smell parts of your body (never/sometimes/often)? Which parts? Why?	Often (1), sometimes (0.5), never (0)

Environment-related olfactory contexts		
Item 3 – Senses in nature	*When you walk in nature, what do you prefer (rank from 1 to 4: touching, smelling, watching, listening)?*	*Smelling is placed first or second (1), third (0.5), fourth (0)*
Item 4 – Yesterday odors	Do you remember odors you smelled yesterday (food odors not acceptable)? Which ones?	Several odors are cited (1), one odor is cited (0.5), the child does not remember (0)
Item 5 – Odors sought when sad	Are there odors you like smelling when you feel sad? Which ones?	Several odors are cited (1), one odor is cited (0.5), no odor is cited (0)
Item 6 – Treasured odorous objects	Are there things you treasure just because they smell very good? Which ones?	Several objects are cited (1), one object is cited (0.5), no object is cited (0)
Item 7 – Outside odors	Imagine there were no odors outside anymore: would you not care/would it bother you/would it suit you – a little/a lot?	It annoys or suits the child a lot (1), a little (0.5), the child does not care about it (0)
Item 8 – Smelling school tools	Do you happen to smell your school things (never/sometimes/often)? Which ones?	Often (1), sometimes (0.5), never (0)
Item 9 – Odor in cars	In your parents' car, does it smell something or nothing? Do you love/like/not care/not like this odor /feel ill?	The child loves the car's odor or feels ill (1), likes or does not like it (0.5), does not care about it (0)
Item 10 – Odor of bathroom objects	Please list objects of your bathroom (up to 8). Which ones smell of something?	> 2/3 of the objects are odorous (1), between 1/3 and 2/3 (0.5), <1/3 (0)
Item 15 – Tobacco smell	Imagine someone is smoking next to you. Do you love/like/not care/not like/hate this odor?	The child loves or hates the odor (1), likes or does not like it (0.5), does not care about it (0)

Reference

1 Ferdenzi, C., Coureaud, G., Camos, V. & Schaal, B. Human awareness and uses of odor cues in everyday life: Results from a questionnaire study in children. *International Journal of Behavioral Development* **32**, 422-431, doi:10.1177/0165025408093661 (2008).

Supplementary Table S3. Odor Exposure Inventory. Listed in the order of appearance in the survey are the individual items, their wording, their response format and, where applicable, the way they were treated in analysis. Next, median and median absolute deviation (MAD) (for frequency/strength ratings) or mode and relative frequency (for binary-response items) are shown. Items excluded from the categorical PCA analysis are given in grey.

Survey Item	Item Wording	Response format	Analysis Item	Median (MAD)	Mode (%)
1 Childcare	(a) Does your child actively participate in taking care of his or her younger sibling (changing diapers, bathing, changing clothes, feeding).	(a) Yes (1)/no (0)	Frequency recorded separately for each activity, from which a median frequency was calculated.		(a) No (68%)
	(b) If so, please rate how often.	(b) Less than once a month (1), about once a month (2), several times a month (3), about once a week (4), several times a week (5), every day (6), several times a day (7)		(b) 1 (0)	
2 Frequency of use of cosmetic products	Please rate how often your child uses any of the following cosmetic products: shower and bath products; body care products; facial care products; antiperspirants, deodorants, and fragrance; children's decorative cosmetics; hair cosmetics; dermatological treatment products.	Less than once a month (1), about once a month (2), several times a month (3), about once a week (4), several times a week (5), every day (6), several times a day (7)	Frequency recorded separately for each product category, from which a median frequency was calculated.	3 (.9)	
3 Strength of scent of cosmetic products	Please rate how strongly scented are the products used (a) by your child and (b) by yourself.	Little to no scent (1), weakly scented (2), moderately scented (3), strongly scented (4), extremely strongly scented (5)	Strength of scent recorded separately for (a) and (b), from which a median frequency was calculated.	2 (1)	
4 Participation in meal preparation and cooking	Please rate how often your child participates in meal preparation or cooking.	Less than once a month (1), about once a month (2), several times a month (3), about once a week (4), several times a week (5), every day (6), several times a day (7)	Due to low frequency (N = 3), several times a day (7) recoded to every day (6).	4 (1)	

Survey Item	Item Wording	Response format	Analysis Item	Median (MAD)	Mode (%)
5 Foreign cuisines, exotic or unusual foods	Do you like to try foreign cuisines, exotic or unusual foods and dishes?	Yes (1)/no (0)			No (71%)
6 Seasoning	Do you use various spices and herbs to season your home-cooked meals?	Yes (1)/no (0)			Yes (63%)
7 Strength of aroma	Please rate the strength of aroma of your home-cooked meals.	Little to no aroma (1), weak (2), moderate (3), strong (4), extremely strong (5)		2 (1)	
8 Herbalism	Do you grow, gather and/or dry herbs at home?	Yes (1)/no (0)			No (70%)
9 Home processing of seasonal produce	Do you bottle, pickle, ferment, etc. seasonal produce?	Yes (1)/no (0)			Yes (56%)
10 Home baking	Do you or another member of your household like to bake?	Yes (1)/no (0)			Yes (69%)
11 Feasts	Do you hold or attend feasts on the occasion of hog-killing, deer hunts, etc.?	Yes (1)/no (0)			No (87%)
12 Household chores	Please rate how often your child participates in the following household chores: washing up;	Less than once a month (1), about once a month (2), several times a month (3), about once a week (4),	Frequency recorded separately for each activity, from which a median frequency was calculated.	1 (1)	

Survey Item	Item Wording	Response format	Analysis Item	Median (MAD)	Mode (%)
	laundry and ironing; bathroom, window, floor, etc. cleaning; hoovering, dusting; waste sorting and disposal; fireplace or stove lighting and cleaning; gardening and farming.	several times a week (5), every day (6), several times a day (7)			
13 Strength of scent of household products	Please rate the how strongly scented are the household cleaning products you use at home.	Little to no scent (1), weakly scented (2), moderately scented (3), strongly scented (4), extremely strongly scented (5)		3 (1)	
14 Home and car fragrance	Do you use on a regular or long-term basis any of the following scented products at home or in your car: air fresheners; aroma lamps; scented candles and sticks; Potpourri; fragrant plants; car air freshener?	(a) Yes (1)/no (0)			Yes (65%)
		(b) Check all that apply.	Number of products selected. Due to low frequency (N = 4), 4 items checked recoded to 3.	1 (1)	
15 Workshop, garage, and garden	Is your child exposed on a regular or long-term basis to any of the following odor sources, whether because of your home business, hobby or do-it-yourself activities: wood; paint, glue, etc.; petrol, motor oil, etc.; building materials; smoke; hay, compost, manure, etc?	(a) Yes (1)/no (0)			Yes (54%)
		(b) Check all that apply.	Number of odor sources selected. Due to low frequency (N = 1), 5 items checked recoded to 4.	1 (1)	
16 Parental hobbies	Do you pursue any of the following hobbies: wood carving, pottery, sculpture, etc.; cooking and baking; gardening; fishing; travelling; farming; floristry; motorsport; horse riding; oil painting; perfumery, aromatherapy	(a) Yes (1)/no (0)			Yes (88%)
		(b) Check all that apply.	Number of hobbies selected. Due to low frequency (N = 1 and 2, respectively), 7 and 6 items checked recoded to 5, respectively.	2 (1)	

Survey Item	Item Wording	Response format	Analysis Item	Median (MAD)	Mode (%)
17 Pets and small domestic animals	Do (i) you yourselves or (ii) someone you and your child visit frequently keep pets or small domestic animals? Which ones?	(a) Yes (1)/no (0)<br><br>(b) If yes, list all that apply.	Due to low response variability in (ii), only the number of pets and small domestic animals kept at home (i) were entered in analysis (see Materials and Methods for details).<br>Due to low frequency (N = 4), 4 animal species listed recoded to 3.	1 (1)	Yes (51%)
18 Odor impregnation	Do you or your partner sometimes come home impregnated with odor, whether due to your profession or leisure activities?	Yes (1)/no (0)			No (86%)
19 Environment	(a) Are there any objects in the vicinity of your home which are, even occasionally, a source of strong odor, whether pleasant of not (busy road, industrial or agricultural facility, dumping ground, restaurant, etc.)?	(a) Yes (1)/no (0)			No (69%)
	(b) If yes, please rate the strength of odor in the vicinity of your home.	(b) Little to no odor (1), weak (2), moderate (3), strong (4), extremely strong (5)	Not analyzed due to small number of responses and low response variability.	2 (1)	
20 Odor naming	Do you teach your child to recognize and name various odors and their sources?	Yes (1)/no (0)			Yes (57%)
21 Attention to odors	Do you draw your child's attention to odors, pleasant or otherwise, of people, things, or places?	Yes (1)/no (0)			Yes (71%)

Survey Item	Item Wording	Response format	Analysis Item	Median (MAD)	Mode (%)
22 Smoking at home	Do you or a family member smoke at home?	Yes (1)/no (0)			No (83%)
23 Strength of odor from smoking	Please rate the strength of odor from smoking, whether from your own or neighboring households.	Little to no odor (1), weak (2), moderate (3), strong (4), extremely strong (5)	Excluded from analysis due to low response variability (see Materials and Methods for details)	1 (0)	
24 Breastfeeding	Has your child been breastfed (for a minimum of 6 months)?	Yes (1)/no (0)			Yes (68%)
